# Exploratory metabolomic analysis based on UHPLC-Q-TOF-MS/MS to study hypoxia-reoxygenation energy metabolic alterations in HK-2 cells

**DOI:** 10.1080/0886022X.2023.2186715

**Published:** 2023-05-29

**Authors:** Xiaoyu Yang, Ailing Kang, Yuanyue Lu, Yafeng Li, Lili Guo, Rongshan Li, Xiaoshuang Zhou

**Affiliations:** a Department of Microbiology and Immunology, School of Basic Medicine, Shanxi Medical University, Jinzhong, China; b Department of Nephrology, Shanxi Provincial People’s Hospital (Fifth Hospital) of Shanxi Medical University, Taiyuan, China; c Department of Core Laboratory, Shanxi Provincial People’s Hospital (Fifth Hospital) of Shanxi Medical University, Taiyuan, China; d Shanxi Provincial Key Laboratory of Kidney Disease, Shanxi Provincial People’s Hospital (Fifth Hospital) of Shanxi Medical University, Taiyuan, China

**Keywords:** Metabolomics, acute kidney injury, metabolic reprogramming, glycolysis, metabolic pathways

## Abstract

**Purpose:**

Renal ischemia-reperfusion injury(IRI)is a major cause of acute kidney injury(AKI), the injury and repair of renal tubular epithelial cells play an important role in the pathological process of IR-AKI. Metabolomics was used to detect cell metabolism alterations and metabolic reprogramming in the initial injury, peak injury, and recovery stage of human renal proximal tubular cells (HK-2 cells) to provide insights into clinical prevention and treatment of IRI-induced AKI.

**Methods:**

An *in vitro* ischemia-reperfusion (H/R) injury and the recovery model of HK-2 cells were established at different times of hypoxia/reoxygenation. Comprehensive detection of metabolic alterations in HK-2 cells after H/R induction by nontarget metabolomics. Interconversion of glycolysis and fatty acid oxidation (FAO) in HK-2 cells after H/R induction was examined by western blotting and qRT-PCR.

**Results:**

Multivariate data analysis found significant differences among the groups, with significant changes in metabolites such as glutamate, malate, aspartate, and L-palmitoylcarnitine. Hypoxia-reoxygenated HK-2 cells are accompanied by altered metabolisms such as disturbance of amino acid and nucleotide metabolism, dysregulation of lipid metabolism, increased glycolysis, and metabolic reprogramming, which manifests as a shift in energy metabolism from FAO to glycolysis.

**Conclusion:**

The development of IRI-induced AKI in HK-2 cells is accompanied by the disturbance of amino acid, nucleotide, and tricarboxylic acid cycle metabolism and specifically metabolic reprogramming of FAO to glycolytic conversion. The timely recovery of energy metabolism in HK-2 cells is of great significance for treating and prognosis IRI-induced AKI.

## 1. Introduction

Acute kidney injury (AKI) is a common clinical syndrome characterized by high clinical mortality and prevalence, defined as a rapid decline in renal function that can eventually lead to acute renal failure (ARF) [1]. The global prevalence of AKI ranges from <1% to 66% [2]. Studies have reported that AKI occurs in approximately 10–15% of hospital admissions, and its incidence in intensive care is over 50% [3]. Despite the significant improvements in the prevention and treatment of AKI, the mortality and morbidity rates remain high, so an in-depth study of the mechanisms underlying the development of AKI is important for the prognosis [2].

Renal ischemia-reperfusion injury (IRI) is a major contributor to AKI, resulting in acute tubular cell necrosis [4]. As an important bearer of reabsorption function, renal tubular cells require a large energy supply and are more energy-dependent than other renal cells, which may partially explain why renal tubular epithelial cells are more prone to injury than other renal intrinsic cells during ischemia or hypoxia in the kidney [5,6]. This prompts comprehensive monitoring of metabolic pathways and alterations in energy metabolism in IR-AKI. Metabolomics, as the closest phenotypic histological technique, can be targeted or untargeted to determine low molecular weight endogenous metabolites in a given cell, tissue, or biological fluid, and its metabolite changes can be used as an accurate predictor in response to metabolic processes [7]. Compared with other biological samples, cell metabolomics has a unique advantage because cells are cultured in a uniform and strict environment, so they have low biological differences and low ethical issues, providing valuable information that is easier to interpret [8].

Ultra-high-performance-liquid chromatography-quadrupole time-of-flight mass spectrometry (UHPLC-Q-TOF-MS/MS) were used to detect all metabolites without complicated sample derivation, which has high selectivity, specificity and accuracy, and can reflect the disorder of intracellular metabolism level to the greatest extent [9].

In this study, metabolomic analysis of human renal proximal tubular cells (HK-2 cells) was performed by UHPLC -Q-TOF-MS/MS, aiming to investigate for the first time the dynamic metabolic changes of HK-2 cells induced by the different times of reoxygenation after hypoxia *in vitro* model. It was used to simulate the initial injury, peak injury, and injury recovery phases after ischemia-reperfusion injury to provide insights into the metabolic changes and clinical prevention and treatment of AKI.

## 2. Materials and methods

### 2.1. Cell culture and cell hypoxia/reoxygenation (H/R) model

HK-2 cells were from Procell Life Science & Technology Co., Ltd (Procell, China, CL-0109). HK-2 cells were grown in Dulbecco’s modified Eagle’s medium (DMEM) supplemented with 10% fetal bovine serum (FBS) and 1% penicillin/streptomycin (P/S) at 37 °C with 100% humidity in 5% CO_2_.

Hypoxia condition was induced by 94% N_2_, 5% CO_2_, and 1% O_2_ and cells were cultured in DMEM medium without FBS and glucose for 24 h. After that, the medium was refreshed again with PBS, and the plates were placed into a normoxic cell incubator (5% CO_2_ and 95% air) for 3 h, 12 h, and 24 h with a complete culture medium. Control cells were incubated in a complete culture medium in a normoxic cell incubator (5% CO_2_ and 95% air).

### 2.2. Quantitative real-time PCR

Total RNA was extracted from HK-2 cells using TRIzol (Mei5 Biotechnology, Beijing, China) and reverse transcribed to cDNA by using a cDNA reverse-transcription kit (Mei5 Biotechnology, Beijing, China). Real-time quantification was then performed by using an SYBR Green PCR Kit (Mei5 Biotechnology, Beijing, China). The relative expression of genes was determined by a Bio-Rad CFX96 Real-Time PCR System (CFX96, Bio-Rad, USA). Gene expression was calculated with the 2-ΔΔCT method. The analysis was performed with Microsoft Excel and GraphPad Prism9. The sequences of the primers are listed as follows in Table 1.

**Table 1.** Primer sequences for real-time PCR.

Gene	Forward primer (5’ to 3’)	Reverse primer (5’ to 3’)
TNF-α	AGCCCTGGTATGAGCCCATCTATC	TCCCAAAGTAGACCTGCCCAGAC
IL-6	CACTGGTCTTTTGGAGTTTGAG	GGACTTTTGTACTCATCTGCAC
IL-1β	GCCAGTGAAATGATGGCTTATT	AGGAGCACTTCATCTGTTTAGG
HK2	CGACAGCATCATTGTTAAGGAG	GCAGGAAAGACACATCACATTT
GLUT1	GATGAAGGAAGAGAGTCGGCAGATG	CAGCACCACAGGCGATGAGGATG
Cpt1α	GATTTCCATTCCTTCCCATTCG	CTCGTATGTGAGGCAAAACTTG
PGC1α	CAGAGAGTATGAGAAGCGAGAG	AGCATCACAGGTATAACGGTAG
GAPDH	CAGGAGGCATTGCTGATGAT	GAAGGCTGGGGCTCATTT

### 2.3 Annexin V-FITC/PI assay for apoptosis detection

According to the manufacturer’s instructions, apoptosis detection was assessed using Annexin V-FITC/PI apoptosis detection kit (meilunbio, China, MA0220). HK-2 cells were seeded into 6-well plates at a density of 2 × 10^5^ cells/well. After overnight culture, the cell H/R model was established. Pancreatic enzyme without EDTA was used to digest the cells and centrifuged the cells then washed with PBS, the cells were collected centrifugally. 1 × Binding buffer solution was added into cell precipitation, and the concentration of cells was suspended to reach 1 × 10^6^ cells/ml. 100 μl (cell number was 1 × 10^5^ cells) was absorbed into the upper sample tube and added with 5 μl Annexin V-FITC and 5 μl PI. The mixture was gently shaken and mixed and incubated at room temperature for 15 min in the dark. After incubation, each tube was added with 400 μl 1 × Binding buffer working solution, check on the machine within an hour and the flow data was analyzed by FlowJo analysis software.Annexin V-FITC(-)/PI(-) represent viable cells, Annexin V-FITC(+)/PI(-) represent early apoptotic cells and Annexin V-FITC(±)/PI(+) represent late apoptotic/necrotic cells.

### 2.4. Metabolomics analysis

#### 2.4.1. Sample collection

There were at least six replicates per group for cellular metabolomics, with one extra for cell counting. The old culture medium was removed, and the cells were washed two or three times with cold PBS. Then collected by cell scraper for the last time. The cell suspensions were collected by centrifugation and registered the number of cells per group by the sample for cell counting and subsequent normalization of the data. The PBS was completely removed, and the cell precipitate was snap-frozen in liquid nitrogen and transported to Shanghai Applied Protein Technology Co., Ltd.

#### 2.4.2. LC-MS/MS analysis

The samples were separated using an Agilent 1290 Infinity LC Ultra High-Performance Liquid Chromatography System (UHPLC) HILIC column. The primary and secondary spectra of the samples were collected using an AB Triple TOF 6600 mass spectrometer and analyzed using electrospray ionization (ESI) positive and negative ion modes, respectively.

#### 2.4.3. Quality control information and data processing

Quality control (QC) samples were prepared by pooling 10 μl of each sample and analyzed together with the other samples. The QC samples were inserted in samples regularly and analyzed to monitor the stability and repeatability of instrument analysis.

In the extracted ion features, only the variables having more than 50% of the nonzero measurement values in at least one group were kept. Compound identification of metabolites was performed by comparing of accuracy m/z value (<10 ppm), and MS/MS spectra with an in-house database (Shanghai Applied Protein Technology) established with available authentic standards.

### 2.5. Western blot analysis

Proteins of HK-2 cells were extracted in RIPA lysis buffer containing PMSF. After determining the protein concentration by the BCA method, the protein samples were mixed 1:4 with 5× loading buffer and boiled at 100 °C for 5 min. Depending on the amount of target protein, samples containing equal amounts of protein were electrophoresed through a 10% SDS-PAGE gel and transferred to a PVDF membrane (Millipore Corp.) at 200 V. Skim milk (5%) was used to block nonspecific binding site and the membranes were then incubated overnight at 4 °C with the following primary antibodies: hexokinase 2 rabbit polyclonal antibody (1:10,000, Cat#22029-1-AP, Proteintech), CPT1A rabbit polyclonal antibody (1:20,000, Cat#15184-1-AP, Proteintech), GAPDH rabbit pAb (1;10000, Cat#AC001, ABclonal). After 3 washes in TBST, incubate with HRP-coupled affinity-pure goat anti-rabbit IgG (H + L) (1:10000, Cat#BA1054, Boster) in 5% skim milk for 1 h at room temperature. The membranes were washed 3 times. Protein bands were detected by enhanced chemiluminescence (ECL, Boster) and quantified by normalizing them to GAPDH levels using ImageJ software, Microsoft Excel and GraphPad Prism9.

### 2.6. Statistical analysis

The metabolomics data after sum-normalization was subjected to multivariate data analysis, the data were analyzed by R-packet (ropls), including Pareto-scaled principal component analysis (PCA) and orthogonal partial least-squares discriminant analysis (OPLS-DA). The 7-fold cross-validation and response permutation testing were used to evaluate the robustness of the model. The variable importance in the projection (VIP) value of each variable in the OPLS-DA model was calculated to indicate its contribution to the classification.

The data of the study were presented as mean ± SD. Multiple group comparisons were performed using an analysis of variance (ANOVA), *p* < 0.05 was considered a statistically significant difference. Statistical analyses were performed by GraphPad Prism9.

## 3. Results

### 3.1. Construction of an in vitro model of injury and recovery due to ischemia-reperfusion

**Figure 1.**
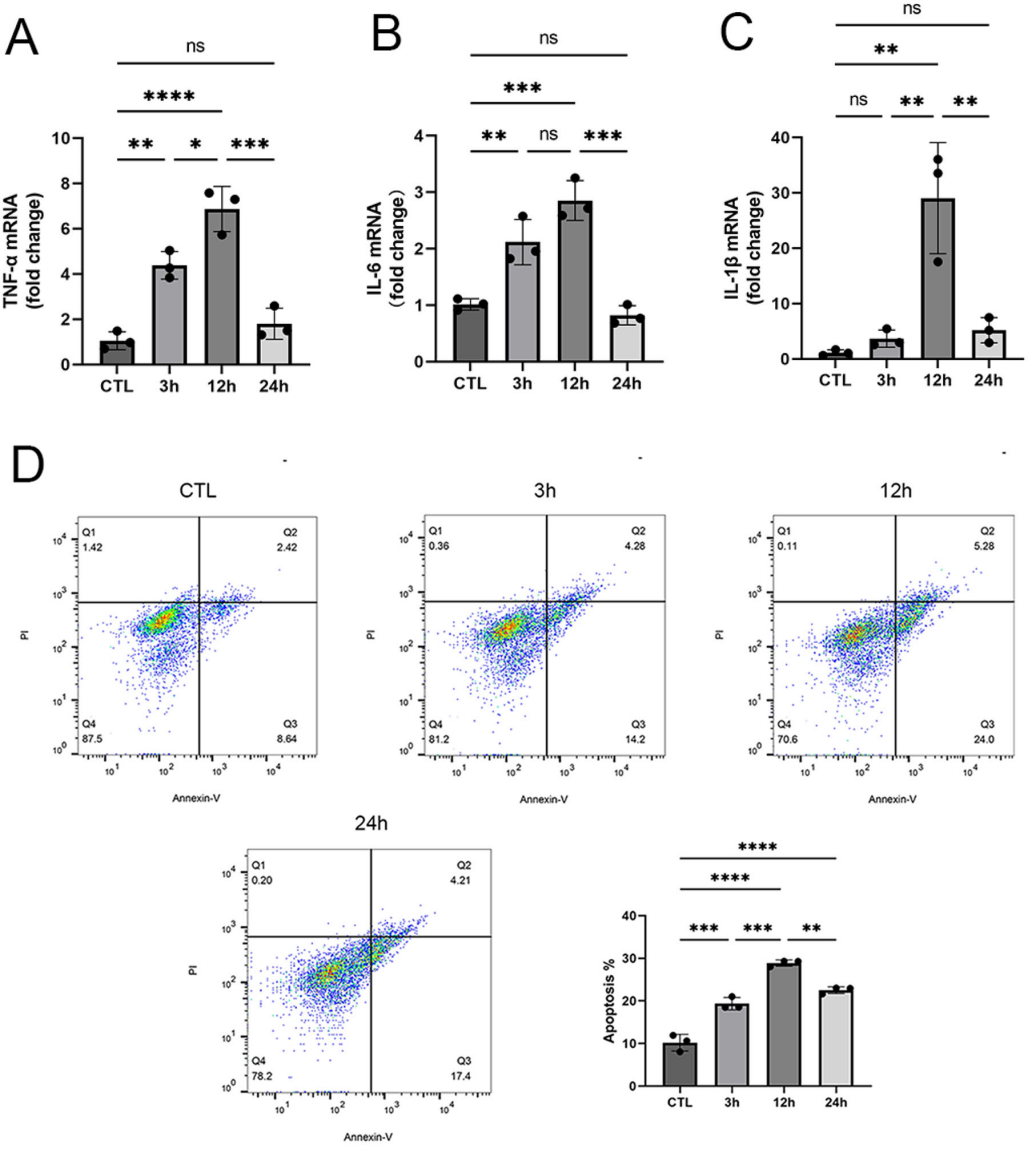
Construction of an *in vitro* model of injury and recovery due to ischemia-reperfusion. **A–C** Changes of TNF-α, IL-1β, and IL-6 mRNA expression at different times of reoxygenation. **D** Flow-through Annexin-FITC/PI double-staining assay to detect apoptosis at different times of reoxygenation. Data are expressed as mean ± SD and *p* < 0.05 was considered statistically significant (*p < 0.05, **p < 0.01, ***p < 0.001, ****p < 0.0001, nsP > 0.05).

Gene expression of inflammation-related factors (TNFα, IL-6,1L-1β) increased at 3h of reoxygenation, was most severe at 12h, and gradually recovered at 24h of reoxygenation (Figure 1(A–C)). Total apoptotic cells consist of early apoptotic cells and late apoptotic cells. The total apoptotic rate increased after hypoxia reoxygenation and was dominated by early apoptotic cells, and the total apoptotic rate and early apoptotic rate were highest at 12-h reoxygenation (Figure 1(D)). It can be seen that this model provides a recoverable model of cellular hypoxia-reoxygenation injury, allowing analysis of metabolic changes during the initial injury, peak injury, and recovery phases.

### 3.2. The changes of energy metabolism in cellular by metabolomics

#### 3.2.1. Results overview

**Figure 2.**
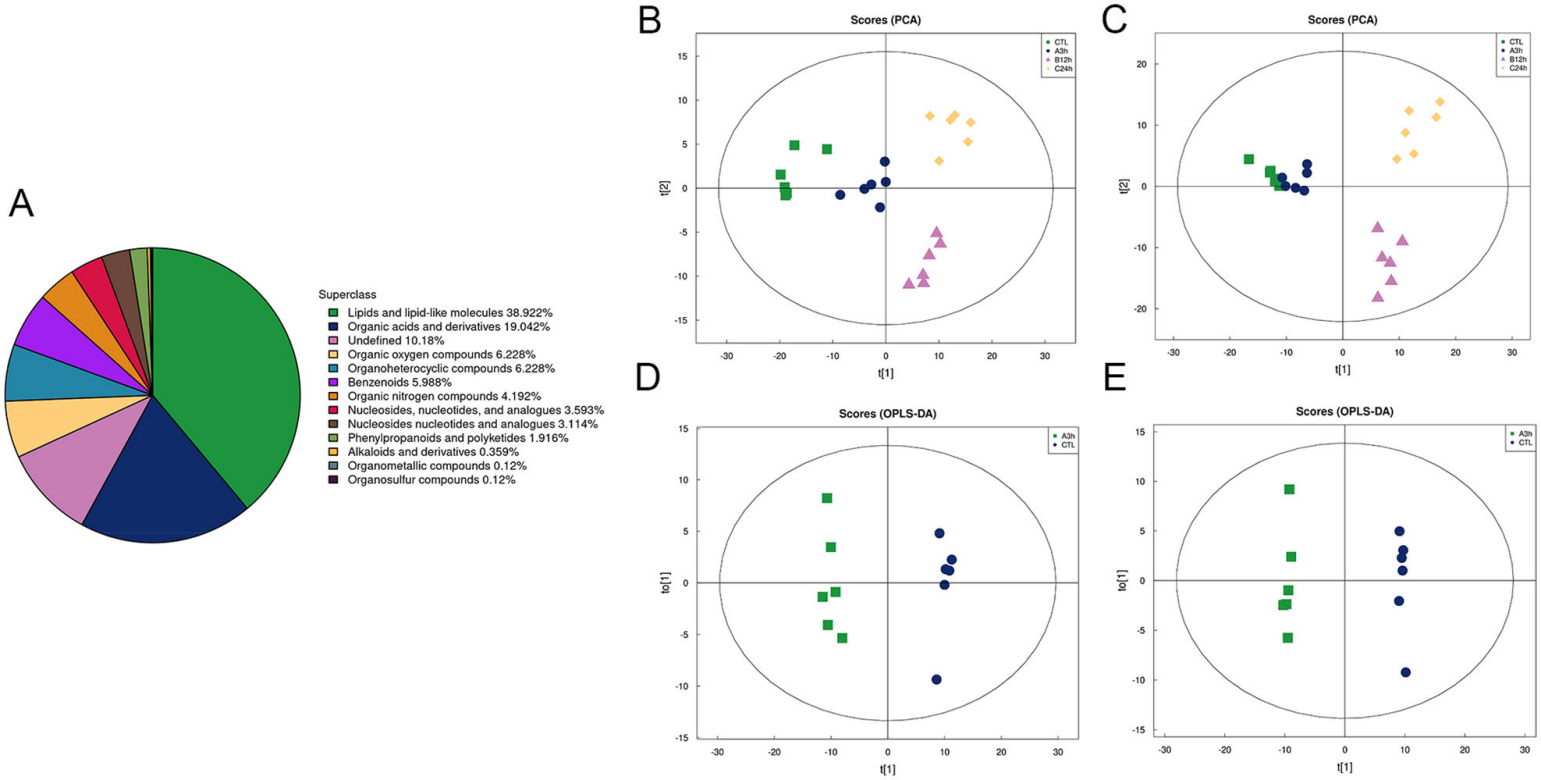
Metabolomics overview and multidimensional statistical Analysis. **A** Several identified metabolites as a percentage of each chemical composition. **B, C** Positive and negative ionization mode PCA score chart. **D, E** 3h vs CTL Positive and negative ionization mode OPLS-DA score chart.

A total of 831 metabolite peaks (513 in positive and 318 in negative ionization mode respectively) were detected, including 13 primary and 63 secondary metabolites. The proportion of primary metabolites (Figure 2(A)) in each chemical classification. The results showed that lipids and lipidoid molecules accounted for the largest proportion (38.992%), followed by organic acids and their derivatives (19.042%), and nucleosides, nucleotides and their analogs accounted for 6.707%.

#### 3.2.2. Multidimensional statistical analysis

The PCA score plot (Figure 2(B,C)) showed that the samples between groups were scattered, and the samples within groups were clustered. The samples from 3h and CTL were separated in the direction of PC1, and those from 12h, 24h, and CTL were separated in the direction of PC1 and PC2. The results of PCA showed the samples of 12h and 24h were different compared with CTL, while the differences between 3h samples and CTL samples were not significant.

The OPLS-DA results of the 3h VS CTL group (Figure 2(D,E)) showed that each comparison group was significantly separated. To prevent model overfitting, 7-fold cross-validation was conducted to validate the OPLS-DA models and drawn a score map (Table 2) Q2 was above 0.9 for all comparison groups, indicating that the models were of high adaptability and predictability.

**Table 2.** OPLS-DA paired model parameters (R2X, R2Y, and Q2) between each comparison group.

OPLS-DA Models	R^2^X(cum)	R^2^Y(cum)	Q^2^(cum)
NEG			
A3h_vs_CTL	0.485	0.990	0.906
B12h_vs_CTL	0.622	0.998	0.980
C24h_vs_CTL	0.643	0.997	0.968
POS			
A3h_vs_CTL	0.509	0.998	0.964
B12h_vs_CTL	0.616	0.997	0.973
C24h_vs_CTL	0.683	0.994	0.974

#### 3.2.3 Identification of differential metabolites

**Figure 3. Screening of differential metabolites and subsequent analysis.**
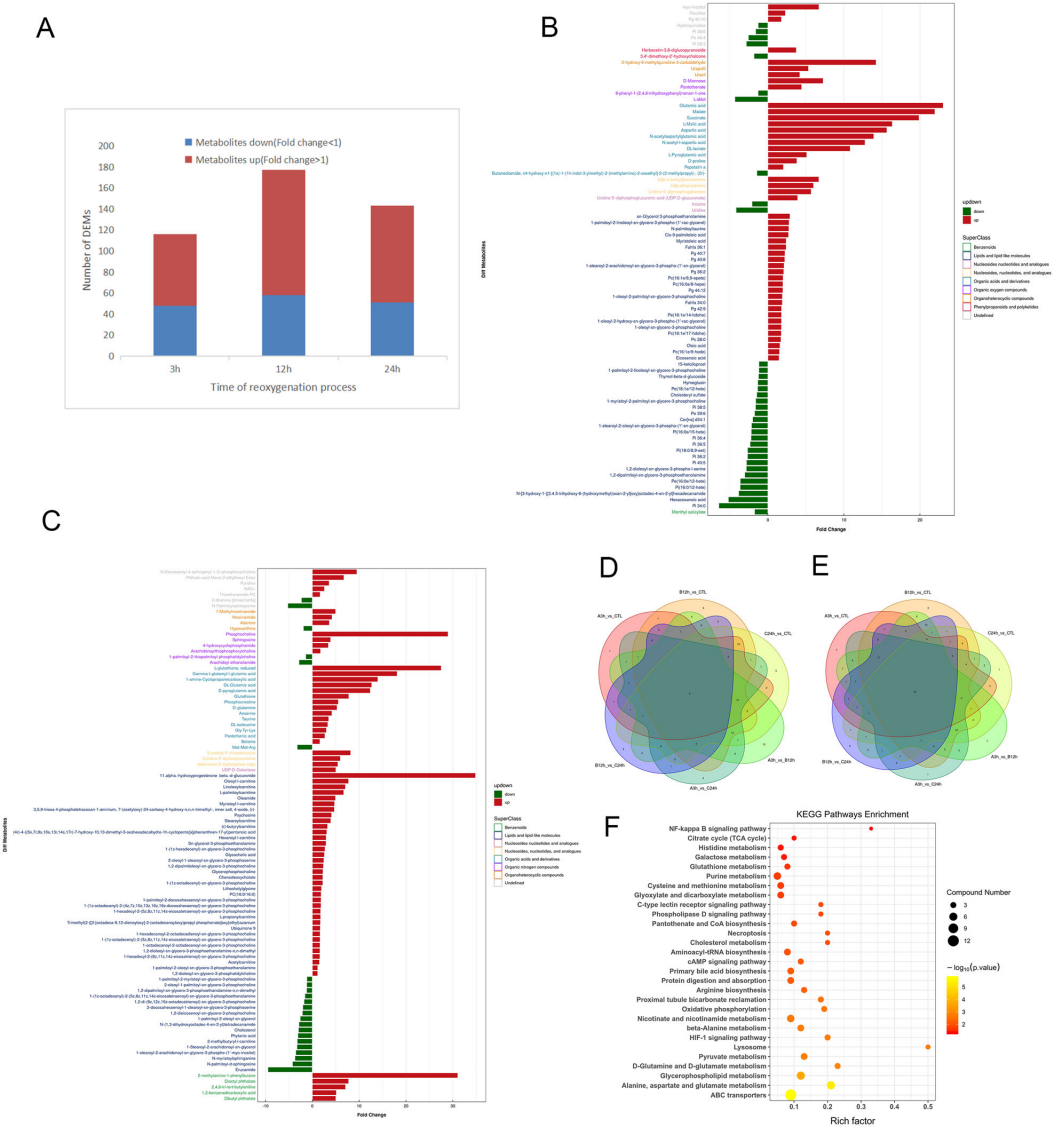
**A** Differential metabolite quantity map at different times. **B, C** Histogram of significant differences in the difference multiplicity of 12h VS CTL in negative and positive ionization mode. **D, E** Venn diagram of differential metabolites in negative and positive ionization. **F** KEGG enrichment pathway bubble map of interest.

Metabolomics usually used strict VIP >1 and *P* value <0.05 as criteria for differential metabolites screening. The number of differential metabolites (Figure 3(A)) showed that the 12h vs CTL group had the largest number of differential metabolites, of which the up-regulated metabolites were predominant, lipids and lipid-like molecules (148/281) were the most in the differential metabolites, followed by organic acids and derivatives (46/281), which showed that lipids and lipid-like molecules and organic acids and derivatives were the metabolites that fluctuated most.

Taking the 12h vs CTL group as an example, the bar graphs (Figure 3(B,C)) showed the variation of significantly different metabolites by superclass fraction in positive and negative ion patterns. The metabolites that changed significantly were glutamate, malate, and aspartate, which will be discussed below. The Venn diagrams (or petal plots) of the differential metabolites (Figure 3(D,E)) for each group comparing. The data suggest that metabolites consistently differ significantly during hypoxia/reoxygenation-induced HK-2 cells injury, including glutamate, Pg 36:2, DL-glutamate, sphingosine, and L-palmitoylcarnitine, which may serve as potential biomarkers.

#### 3.2.4. Pathway enrichment analysis for differential metabolites

KEGG pathway analysis was performed on all differential metabolites to identify the major metabolic pathways involved at each time point. The significant KEGG enrichment pathway bubble map of interest (Figure 3(F)) mainly enriched biosynthetic metabolic pathways such as amino acids, lipids, and organic acids and energy metabolic pathways such as the TCA cycle and oxidative phosphorylation.

### 3.3. Metabolic reprogramming of HK-2 cells in H/R

**Figure 4.**
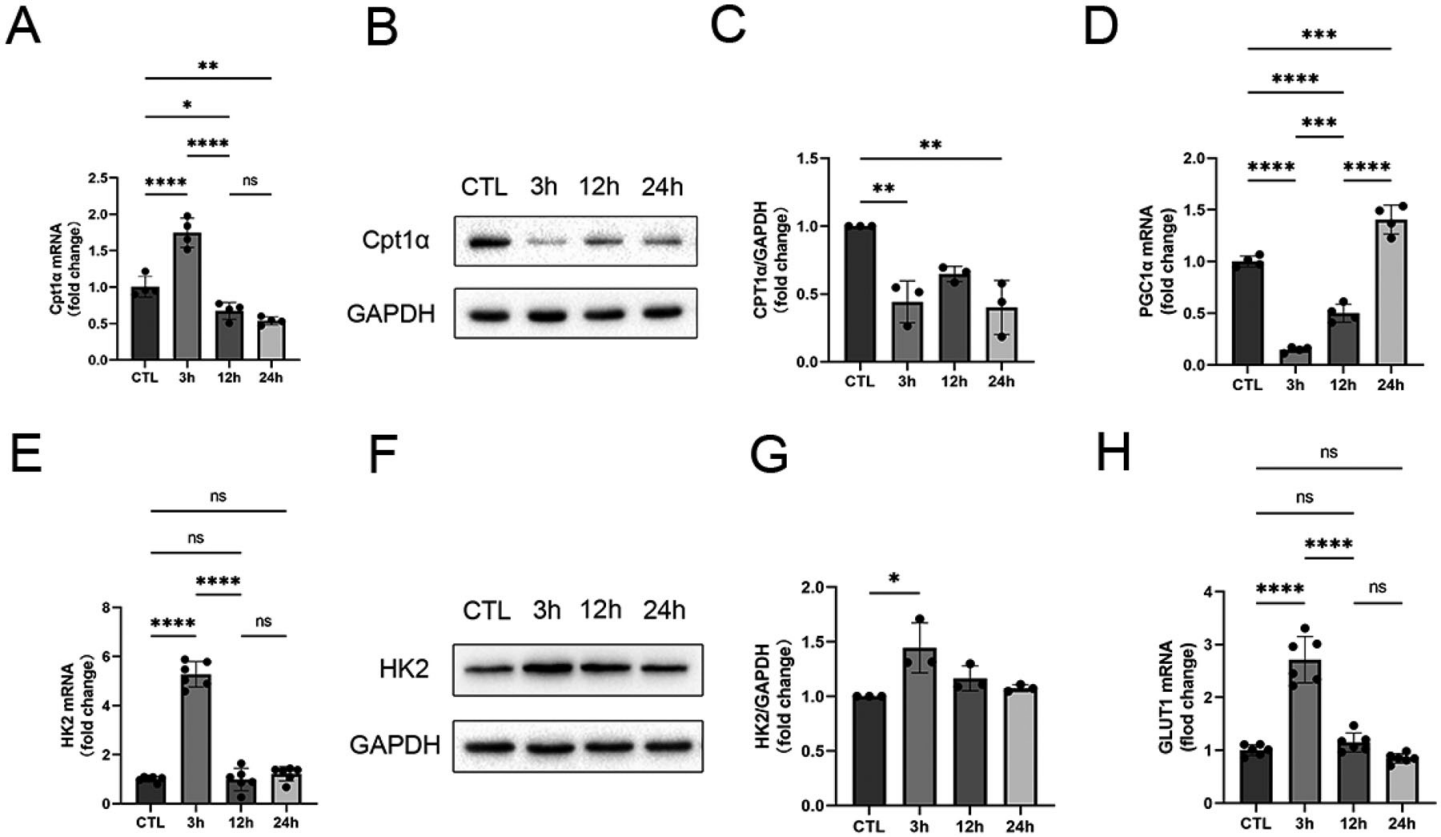
Metabolic reprogramming of fatty acid oxidation to glycolytic transformation in HK-2 cells in H/R. **A-C** qRT-PCR and western blots analysis of Cpt1α. **D** qRT-PCR analysis of PGC1α. **E-G** qRT-PCR and western blots analysis of HK2. **H** qRT-PCR analysis of GLUT1.GAPDH was used as the internal control. Data are expressed as mean ± SD and *p* < 0.05 was considered statistically significant (*p < 0.05, **p < 0.01, ***p < 0.001, ****p < 0.0001, nsP > 0.05).

The mRNA and protein expression levels of carnitine palmitoyl-transferase 1α (Cpt1α), a key enzyme in fatty acid oxidation, decreased (Figure 4(A–C)). In addition, reduced mRNA expression of peroxisome proliferator-activated receptor γ coactivator 1α (PGC1α), a key upstream factor regulating fatty acid oxidation levels (Figure 4(D)). This demonstrated that HK-2 cells have fatty acid oxidation disorders in H/R.

The mRNA and protein expression of hexokinase 2 (HK2), a key enzyme of glycolysis, was significantly increased at 3 h of reoxygenation (Figure 4(E–G)). Gene expression of glucose transporter protein 1 (GLUT1) was also increased (Figure 4(H)). This suggests that the energy metabolism of HK-2 cells may change from fatty acid oxidation to glycolysis at the early stage of injury to compensate for the impaired mitochondrial energy metabolism of HK-2 cells in H/R to adapt to the disease environment [10,11].

## 4. Discussion

### 4.1. Disturbance of amino acid metabolism

**Figure 5.**
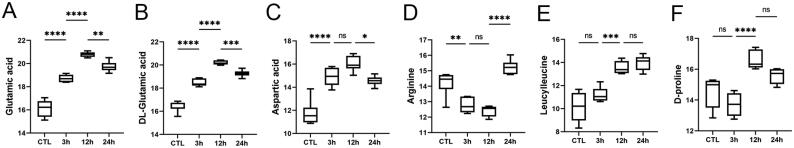
Disturbance of amino acid metabolism. **A–F** Changing trend of representative amino acid. Data were standardized by log2 transformation and expressed as mean ± SD. P < 0.05 was considered statistically significant (*p < 0.05, **p < 0.01, ***p < 0.001, ****p < 0.0001, ns*p* > 0.05).

With the increase in reoxygenation time, the content of most amino acids in HK-2 cells showed an increasing trend and reached a peak at 12h. Notably, the arginine content decreased and reached a trough at 12 h of reoxygenation, in contrast to the trend of the other amino acid contents. Glutamate (Figure 5(A,B)) is one of the kidney’s most important substrates for ammonia production and plays an important role in acid-base homeostasis. HK-2 cells’ mitochondria can oxidize glutamate to α-ketoglutarate, which enters the TCA cycle *via* transamination and is further converted to succinate, which is used to supplement the impaired TCA cycle [12]. Aspartic acid and arginine (Figure 5(C,D)) were involved in the urea cycle and transported NAD + transport into mitochondria with the malate–aspartate shuttle, which was involved in the tricarboxylic acid cycle, glycolysis, and the electron transport chain [13,14]. In the cells of proximal renal tubules, arginine synthetase acts on citrulline to produce arginine [15]. But the trend of arginine is opposite to that of aspartic acid. The probable cause is a decrease in renal arginine production during unilateral ischemia-reperfusion, a change that may facilitate the recovery of low plasma arginine levels after trauma, shock, or vascular surgery [16]. Transcription factor Krüppel-like factor 6 (KLF6) was strongly induced after AKI. KLF6-mediated inhibition of BCAA catabolism can lead to increased BCAA levels such as Leucylleucine (Figure 5(E)) [17]. D-proline accumulates during renal insufficiency, and proline induces oxidative stress and lipid peroxidation in rat kidneys [18,19]. It has been shown that the greatest fluctuations in amino acid metabolism among metabolic pathways were observed in a renal ischemia-reperfusion model, suggesting that amino acid metabolism may be a significant but unnoticed pathway in the development of IR-AKI [20].

### 4.2. Nucleotide metabolism disorder

**Figure 6.**
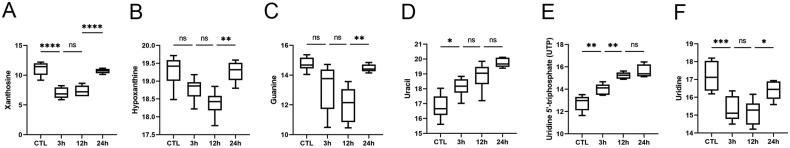
Nucleotide metabolism disorder. **A–F** Changing trend of representative nucleotides. Data were standardized by log2 transformation and expressed as mean ± SD. *P* < 0.05 was considered statistically significant (*p < 0.05, **p < 0.01, ***p < 0.001, ****p < 0.0001, nsP > 0.05).

The increase in purine metabolism is manifested by a significant decrease in xanthine, hypoxanthine, and guanine (Figure 6(A–C)). These ATP metabolites can diffuse freely from proximal renal tubular epithelial cells and may be used in the future as noninvasive markers of altered intracellular ATP metabolism associated with AKI [21]. Uracil and uridine 5′-triphosphate (UTP) were found to increase while their precursor uridine decreased, indicating a possible increase in pyrimidine synthesis in HK2 cells after ischemia-reperfusion injury (Figure 6(D–B)).

### 4.3. Dysregulation of lipid metabolism

**Figure 7.**
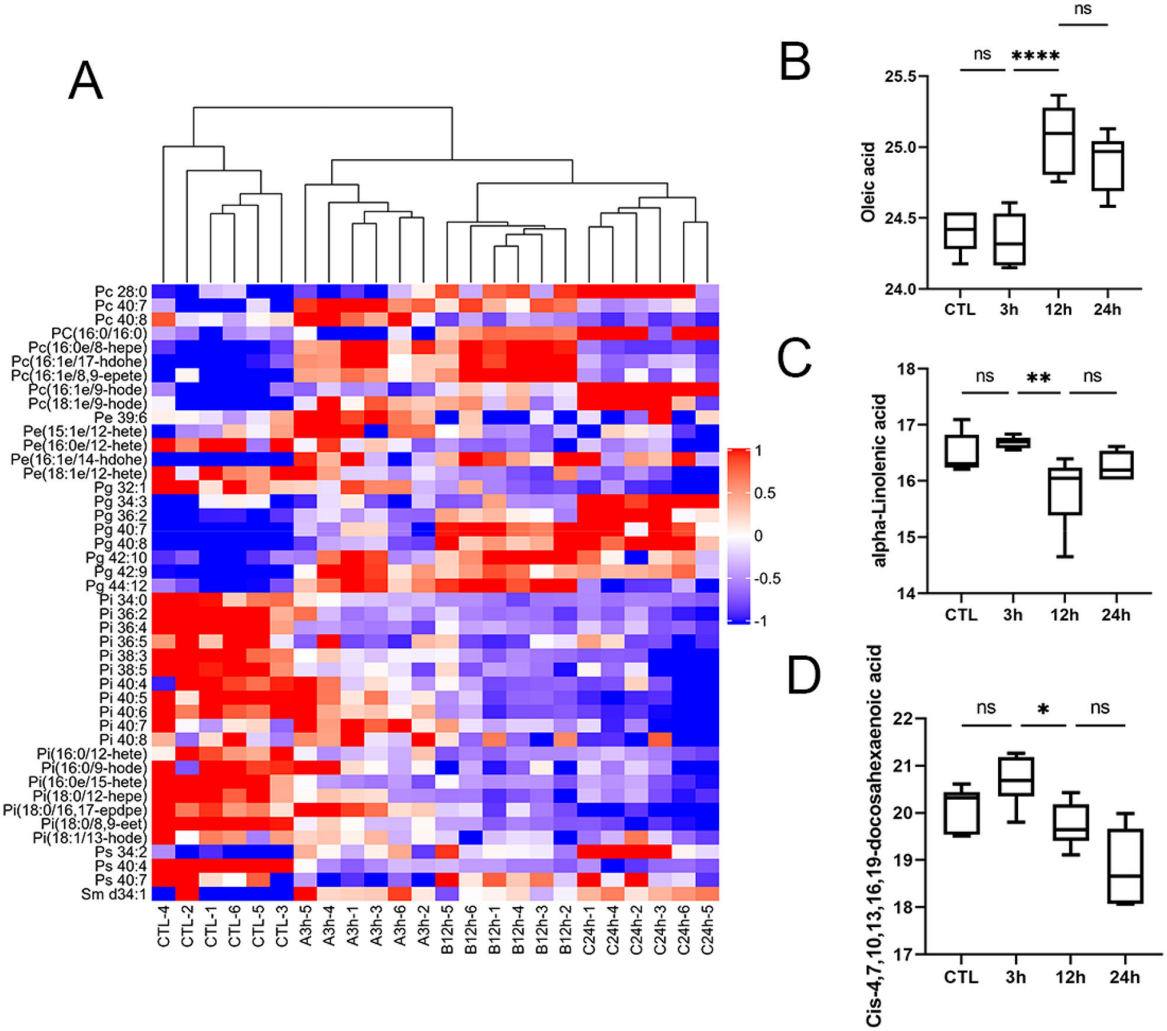
Dysregulation of lipid metabolism. **A** Clustering heat map of membrane lipid components. **B–D** Expression of representative lipids within the group. Data were standardized by log2 transformation and expressed as mean ± SD. *P* < 0.05 was considered statistically significant (*p < 0.05, **p < 0.01, ***p < 0.001, ****p < 0.0001, ns*p* > 0.05).

As part of lipids, glycerophospholipids can be divided into PE.PS, PC, PI, PG, and PA according to the different head groups. Various forms of AKI, including IRI, induce cell membrane instability, leading to lipid dysfunction and abnormal lipid accumulation in the kidney, and it has been documented that such alterations occur in the proximal tubule, including alterations in PE and PC species and levels [22,23]. In our study, the heat map (Figure 7(A)). showed that PC and PG showed an upward trend, and PE and PI showed a downward trend, especially the decline of PI was the most obvious.

Free fatty acids (FFA), also known as non-esterified fatty acids (NEFA), are lipotoxic and consist of oleic acid, palmitic acid, linoleic acid, etc. Intracellular levels of oleic acid are elevated (Figure 7(B)), and its accumulation in the renal cortex has been reported to activate peroxisome proliferator-activated receptors (PPARs) leading to lipid stress and induction of renal injury [24,25]. Polyunsaturated fatty acids (PUFA) undergo auto-oxidation to form oxidized lipids, which a part of them have anti-inflammatory properties [26,27], such as alpha-linolenic acid and docosahexaenoic acid (DHA) derivative (Figure 7(C,D)), were shown to have reduced levels. It has been shown that exogenous DHA supplementation significantly altered the oxylipin levels and improved tubular function after ischemia-induced acute kidney injury [28]. Disturbances in membrane lipid composition and elevated levels of oleic acid, and reduced levels of polyunsaturated fatty acids may suffer from impaired fatty acid oxidation, leading to increased lipotoxicity and inflammation [29,30].

#### 4.4. Glycolysis enhancement

**Figure 8.**
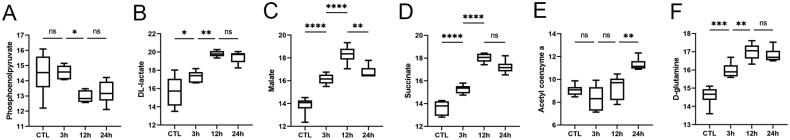
Glycolysis enhancement and replenishing TCA cycle. **A, B** The expression of glycolysis related metabolites. **C-F** The expression of TCA cycle related metabolites. Data were standardized by log2 transformation and expressed as mean ± SD. *P* < 0.05 was considered statistically significant (*p < 0.05, **p < 0.01, ***p < 0.001, ****p < 0.0001, ns*p* > 0.05).

Phosphoenolpyruvate (PEP) (Figure 8(A)) is phosphorylated to pyruvate, which regulates cellular metabolism as the final step in glycolysis to produce ATP. During ischemia, TECs lead to pyruvate depletion and increased lactate levels (Figure 8(B)) in the renal cortex by inducing a shift in the HIF1α subunit from FAO to glycolysis [31]. It has been shown that the glycolytic pathway plays a key role 48 h after reperfusion, and the accumulation of lactate, the end product of anaerobic glycolysis, was observed particularly in urine and renal lysates [32]. Glycolysis and an increasing trend of DL-lactate as an end product of glycolysis peaked at 12h in our study, indicating an early increase in glycolysis.

#### 4.5. Metabolic reprogramming phenomenon

In the normal physiological state, the kidney derives most of its energy from fatty acid oxidation (FAO) rather than glycolysis. In contrast, during the development of AKI, enhanced glycolysis is used to complement the disruption of FAO [33]. This above shift in energy metabolism is similar to the Warburg effect in tumor cells and is also known as metabolic reprogramming of renal tubular epithelial cell [33,34]. It is worth noting that although energy metabolic reprogramming is a protective mechanism for proximal renal tubule cells to adapt to pathological environment, lipid accumulation and lipid toxicity will be caused by fatty acid uptake and oxidation disorders, and sustained glycolysis may lead to kidney injury [29,35,36]. Moreover, such compensation mechanism cannot fully compensate for the loss of energy, and long-term incomplete repair may lead to atrophy of renal tubular epithelial cells, maladaptive repair, and progression to CKD and fibrosis [37–41].

#### 4.6. Replenishing the tricarboxylic acid (TCA) cycle

Succinic acid and malate (Figure 8(C,D)) are involved in energy metabolism as TCA circulating metabolites. Under conditions of ischemia and hypoxia, malate is dehydrated to fumarate, which is reduced to succinate, accumulates to drive reactive oxygen species generation and compensates for the reduced pool of electron donors and carriers, and after reperfusion, succinate accumulated during ischemia is rapidly oxidized [42,43]. Acetyl coenzyme A (Figure 8(E)) acts as a substrate for the tricarboxylic acid cycle, releasing large amounts of energy. In addition, the glutamine enters the TCA cycle, and glutamine (Figure 8(F)) enters the mitochondria to form glutamate, which, as mentioned above, is converted to α-ketoglutarate to replenish the TCA cycle [12,44,45]. This suggests that the kidney produces ATP by altering metabolic substrates to maintain the energy deficit at the time of injury.

### 4.7. Other aspects

**Figure 9.**
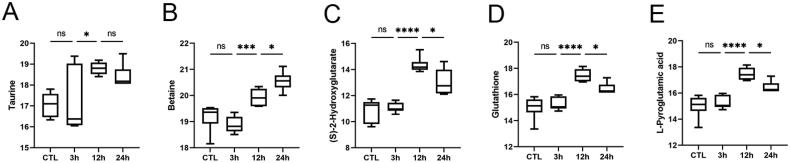
Other representative metabolites. **A, B** Osmotic pressure related metabolite expression. **C** Expression of immune metabolites. **D, E** Oxidative stress related metabolite expression. Data were standardized by log2 transformation and expressed as mean ± SD. *P* < 0.05 was considered statistically significant (*p < 0.05, **p < 0.01, ***p < 0.001, ****p < 0.0001, ns*p* > 0.05).

In addition to this, H/R also affects changes in renal osmotic pressure, manifested as changes in the levels of osmotic substances such as taurine and betaine (Figure 9(A,B)). Taurine levels were reduced in the kidney after ischemia-reperfusion but increased in HK-2 cells, probably in association with Na + transport in proximal tubule cells, and the efflux of taurine in the cells was much slower than the uptake [32,46]. Choline is oxidized in the kidney to betaine, which acts as an osmoprotectant. Betaine in serum is reduced in an experimental model of septic acute kidney injury, while betaine inside the cells in our study was on the rise [47].

S-2-hydroxyglutarate(S-2-HG) (Figure 9(C)) acts as an immunometabolite that links environmental context, through a metabolic-epigenetic axis, to immune fate and function [48]. S-2-HG expression was controlled by hypoxia-inducible factors (HIFs) and showed a dose-dependent nephroprotective effect at the assessed doses [49].

The significant increase in glycolysis also promoted the pathway of pentose phosphate pathway (PPP), which maintains the reduced state of glutathione (GSH) (Figure 9(D)) with uptake by renal tubular epithelial cells, as evidenced by increased levels of GSH. Pyroglutamic acid (Figure 9(E)), a downstream metabolite of glutathione, tends to increase as an antioxidant and thus resists damage from peroxide in the renal tubules [50–52].

## 5. Conclusions

In conclusion, this study provides a UHPLC/Q-TOF-MS/MS based cellular metabolomics that can accurately distinguish metabolites at different reoxygenation times and explore metabolic changes during the initial injury, peak injury and recovery phases of AKI. The changes in metabolites suggest that the development of IRI-induced AKI is accompanied by reprogramming of amino acid, nucleotide and tricarboxylic acid cycle metabolism and conversion of fatty acid oxidation to glycolysis. This suggests that our timely restoration of normal energy metabolism as a strategy to promote renal repair could alter the fate of renal tubular epithelial cells. However, so far, these remain hypotheses that need to be tested *in vivo*.

## Data Availability

All data are included in the manuscript.
